# Acute-onset paraplegia as an unexpected complication under general anesthesia in supine position during abdominal endovascular aneurysm repair: a case report

**DOI:** 10.1186/s40981-021-00447-7

**Published:** 2021-06-02

**Authors:** Atsushi Morio, Hirotsugu Miyoshi, Noboru Saeki, Yukari Toyota, Yasuo M. Tsutsumi

**Affiliations:** Department of Anesthesiology and Critical Care, Hiroshima University, 1-2-3 Kasumi, Minami-ku, Hiroshima, 734-8551 Japan

**Keywords:** Paraplegia, Spinal cord injury, Ischemic spinal cord injury, Endovascular aneurysm repair, Supine position

## Abstract

**Background:**

Acute onset paraplegia after endovascular aneurysm repair (EVAR) is a rare but well-known complication. We here show a 79-year-old woman with paraplegia caused by static and dynamic spinal cord insult not by ischemia after EVAR.

**Case presentation:**

The patient underwent EVAR for abdominal aortic aneurism under general anesthesia in the supine position. She had a medical history of lumbar canal stenosis. After the surgery, we recognized severe paraplegia and sensory disorder of lower limbs. Although the possibility of spinal cord ischemia was considered at that time, postoperative magnetic resonance imaging (MRI) revealed burst fracture of vertebra and compressed spinal cord.

**Conclusions:**

Patients with spinal canal stenosis can cause extrinsic spinal cord injury even with weak external forces. Thus, even after EVAR, it is important to consider extrinsic factors as the cause of paraplegia.

## Background

Acute-onset paraplegia usually results from either a traumatic or an ischemic spinal cord insult. Rapid administration of treatment is essential to avoid permanent spinal cord damage; thus, quick diagnosis of spinal cord injuries is vital. In many cases, spinal cord injuries result from a strong external force; however, patients with spinal stenosis can sustain an extrinsic spinal cord injury even if the external force is weak. Symptoms of traumatic spinal cord injuries vary depending on the part of the spinal cord that is involved; however, paraplegia is usually accompanied by sensory impairment [1–3].

Endovascular aneurysm repair (EVAR) in patients with an abdominal aortic aneurysm is a minimally invasive procedure with unique complications [4]. Paraplegia due to spinal cord ischemia (SCI) is a rare, but well-known complication of EVAR [5–7]. Since the anterior portion of the spinal cord is usually supplied by only one anterior spinal artery, it is especially susceptible to ischemia due to hemodynamic changes caused by EVAR [4]. Anterior SCI is normally characterized by paraplegia without impairment of deep sensation. Here, we report a case of a 79-year-old woman with acute paraplegia after EVAR due to an extrinsic spinal cord injury rather than ischemia.

## Case presentation

**Fig. 1:**
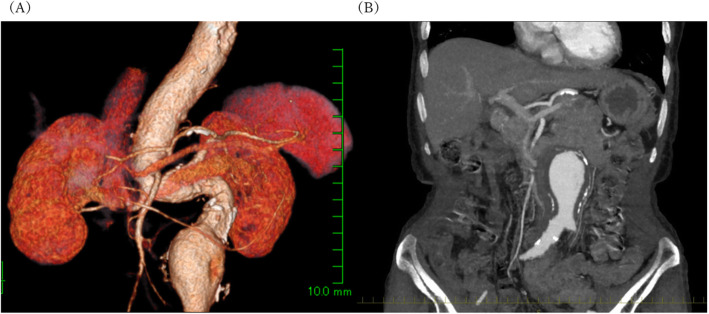
Preoperative computed tomography. **A** An aneurism, 47 mm in diameter, under the renal artery. **B** The aneurism contained a thick intramural thrombus and the lumbar arteries were almost occluded

A 79-year-old woman (height, 137 cm; weight, 40 kg; body mass index, 21.3 kg/m^2^) was diagnosed with an abdominal aortic aneurysm during preoperative computed tomography (CT) for early stage gastric cancer. The aneurism was 47 mm in diameter and was located under the renal artery (Fig. 1A). The iliac artery was patent; however, the lumbar arteries were almost occluded by a thick intramural thrombus (Fig. 1B). Because of the risk of rupture, we planned to perform EVAR. She had a history of cerebral infarction without sequelae, as well as rheumatoid arthritis, pulmonary emphysema, and lumbar spinal stenosis resulting from kyphosis (T12-L2 Cobb angle; 32.2°). Before the surgery, she was inactive because of back pain, although she was able to grasp objects and walk with a cane.

EVAR was performed under general anesthesia, and a stent graft (Endurant II; Medtronic plc, Dublin, Ireland), was placed in the abdominal aortic aneurysm. General anesthesia was induced with propofol (target-controlled infusion [TCI], 3 μg/mL), remifentanil (1 mg/h), and rocuronium (25 mg). Anesthesia was maintained with propofol (TCI, 1.5–2.0 μg/mL; total, 337 mg), remifentanil (1 mg/h), and fentanyl (200 μg). The operative time was 92 min and blood loss was 41 ml; the surgical procedure was performed without any problems and the patient remained hemodynamically stable throughout the operation.

**Fig. 2:**
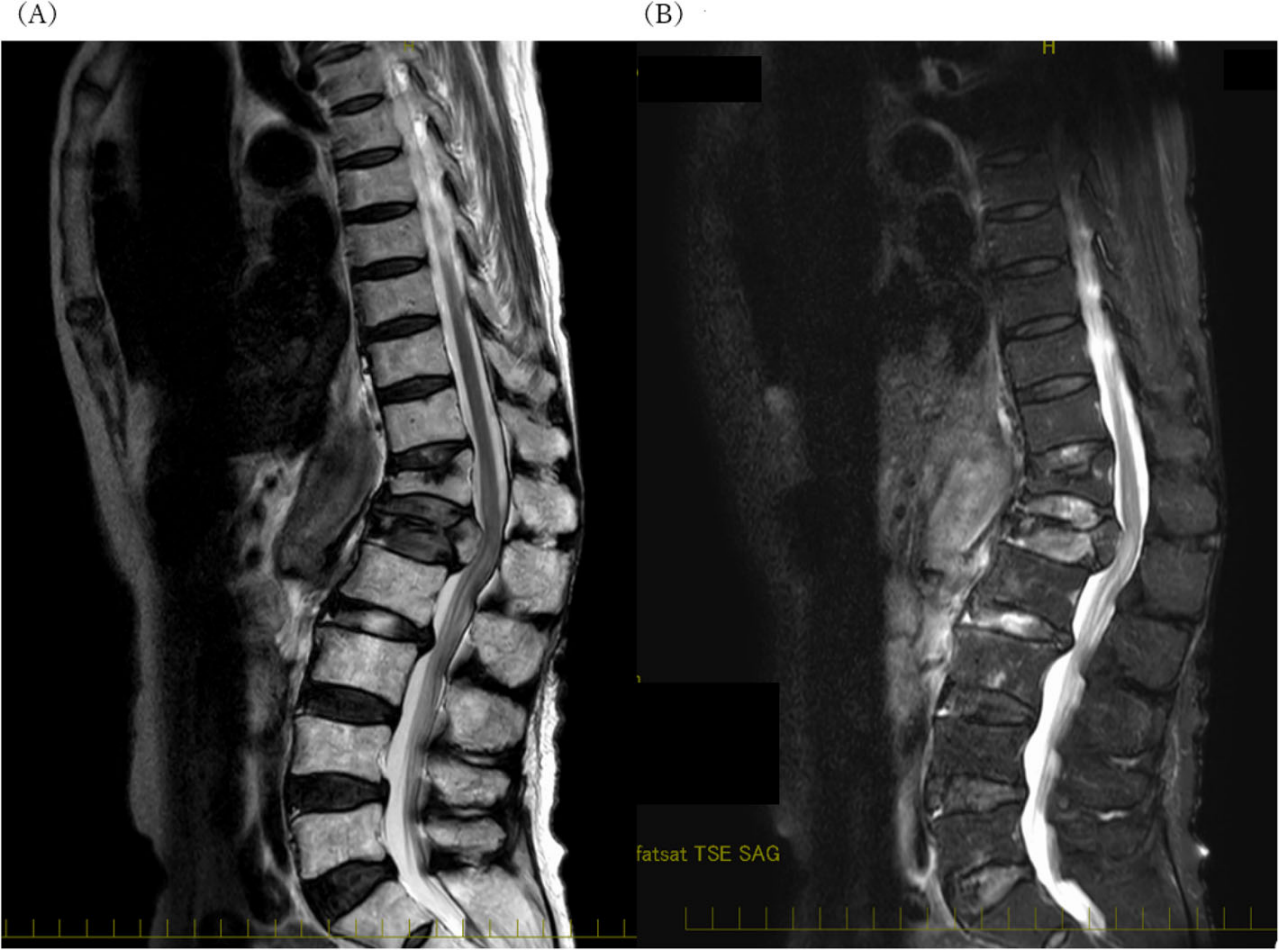
Magnetic resonance imaging on postoperative day 1. **A** A sagittal, T2-weighted image revealed the compressed spinal cord at the L1 level. **B** A sagittal, fat-suppression, T2-weighted image revealed a low intensity of the T12 vertebra and a high intensity of a portion of the L1 vertebra

After the surgery, the administration of anesthetics was interrupted and the patient was extubated. Upon waking, although she was fully conscious and could communicate normally, she could not move her legs at all. Her manual muscle testing (MMT) score was 0. Although the cause of paraplegia was unclear, we considered the possibility of SCI. In addition to the paraplegia, we confirmed that she experienced sensory impairment below L1. She could barely recognize being touched, in addition, the position and passive movement of her knee and ankle joints. In other words, the sensory impairment was both superficial and deep part of her lower limbs, which is atypical of paraplegia after EVAR. Her neurological status was grade B of acute spinal cord injury-Frankel Classification grading system. Subsequently, we performed cerebrospinal fluid drainage at the L3/4 intervertebral space, and a catheter was left in order to decompress the spinal canal and augment perfusion of the spinal cord. Cerebrospinal fluid drainage was conducted at the L3/L4 interspinal space, and the catheter (PORTEX epidural catheter, Smiths medical, Saint Paul, USA) was left in place. After cerebrospinal fluid drainage at pressure management setting of 10 cmH_2_0, the patient’s symptoms improved slightly; most importantly, we confirmed that she could slightly contract the muscles and move her lower limbs a little. At that time, her MMT score was 2. Consequently, she was admitted to the intensive care unit (ICU). In the ICU, we administered intravenously an established treatment for paraplegia due to SCI: naloxone (40 μg/h), methylprednisolone (1000 mg/day), and edaravone (60 mg/day). On postoperative day (POD) 1, magnetic resonance imaging (MRI) revealed compression of the spinal cord at the L1 level, a burst fracture of the L1 vertebra and an old fracture of the T12 vertebra, but no ischemic changes in the spinal cord (Fig. 2A, B). A T2-weighted, fat-suppression image revealed a high signal intensity of the spinal cord at the L1 level, a burst fracture of the L1 vertebra, and an old fracture of the T12 vertebra (Fig. 2B). No ischemia was visible in the spinal cord. These results indicated the possibility of an insult to the spinal cord caused by fractured vertebrae. Hence, we diagnosed the patient with extrinsic spinal cord injury. No surgery was performed at that time, per the patient’s request, although decompression and stabilization of the fractured vertebrae might have further improved the condition. Although the patient experienced sequelae of the injury, she was discharged from the ICU on POD5. Despite rehabilitation in a general patient ward, the patient’s symptoms did not improve dramatically. Her MMT score was almost 2 or 3 and her neurological status was Frankel C; thus, she could move only by wheelchair and needed more rehabilitation. Therefore, she was transferred to a rehabilitation hospital on POD43. As for early stage gastric cancer, it was resected endoscopically, and the patient is still being followed up in the department of gastroenterology.

## Discussion

In the present case, we reported on a patient who developed paraplegia after EVAR. We immediately suspected SCI as the cause; however, postoperative MRI revealed compression of the spinal cord due to a burst fracture of the L1 vertebral body. We determined that static and dynamic compression to the spinal cord was caused by the patient’s supine position and extension of her legs during the operation under general anesthesia. It is important to take account of the possibility of traumatic injury as the cause of postoperative paraplegia, even if it occurs after a minimally invasive procedure such as EVAR. To the best of our knowledge, there have been no reports of paraplegia after EVAR caused by the supine position.

The characteristic adjunct symptom of paraplegia due to SCI after EVAR is superficial but not deep dissociative sensory loss [5–7]. The anterior portion of the spinal cord mainly consists of the pyramidal and spinothalamic tracts, which are responsible for motor function, as well as sensations of pain, temperature, crude touch, and firm pressure. On the other hand, the posterior portion of the spinal cord, consisting of the gracile and cuneate fasciculi, is responsible for position sensation, fine touch, vibration sensation, and two-point discrimination. The spinal cord is usually supplied by one anterior and two posterior spinal arteries. Therefore, although EVAR causes substantial hemodynamic changes in the spinal cord, its posterior portion, which is responsible for deep sensation, is less susceptible than its anterior portion to ischemia. Consequently, the characteristic symptom caused by SCI after EVAR is paraplegia, usually without impairment of deep sensation. In contrast, sensory impairment associated with extrinsic spinal cord injury is more varied, depending on the portion that is damaged [1–3]. In the current case, we confirmed that the patient experienced not only superficial but also deep sensory impairment; however, her symptoms were atypical of SCI due to EVAR. Therefore, when a patient experiences paraplegia after EVAR, extrinsic factors should also be considered to ensure that the correct treatment is started promptly.

With regard to the patient’s medical history, she was unable to walk unassisted and could not sleep well in the supine position due to severe kyphosis. A patient with spinal stenosis may sustain a spinal cord injury when the spine is extended more than usual. Generally, patients undergoing EVAR need to lie in the supine position and extend both legs for puncturing of the femoral artery. In this case, before performing EVAR, we did not confirm whether the patient was able to extend her legs completely and lie in the supine position for a long time. EVAR is often performed in older individuals; furthermore, the prevalence of symptomatic lumbar spinal stenosis is about 10% in that population [1]. Therefore, before performing EVAR in older individuals, it is important to evaluate their spinal cord and to confirm whether they are comfortable in the supine position.

Additionally, there are some possible improvement points as for treatment. First, in this case, the operation was conducted under general anesthesia with muscle relaxant aiming for hemodynamically stable condition. If we chose local anesthesia with mild sedation, we might have confirmed early, or the force on her back may have weakened. Second, the symptom tended to improve slightly; thus, surgery was not performed at that time, per the patient’s request; however, quick MRI examination and decompression and stabilization of fractured vertebrae might have been desired.

In summary, we reported the case of a 79-year-old woman with paraplegia caused by the compression of the spinal cord after EVAR. As the cause of paraplegia after EVAR, it is important to consider the possibility of extrinsic factors, not only ischemia, in patients with spinal problems. If paraplegia is accompanied by impaired sensation in the lower extremities, physical compression of the spinal nerves may be the cause. In such cases, even with post-EVAR paraplegia, we need to perform an immediate MRI examination to determine the cause of the paraplegia

## Data Availability

Not applicable due to patient privacy concern

## Abbreviations

EVAR: Endovascular aneurysm repair
SCI: Spinal cord ischemia
TCI: Target-controlled infusion
MMT: Manual muscle testing
CT: Computed tomography
MRI: Magnetic resonance imaging
POD: Postoperative day
